# Autoreactivity to Glucose Regulated Protein 78 Links Emphysema and Osteoporosis in Smokers

**DOI:** 10.1371/journal.pone.0105066

**Published:** 2014-09-12

**Authors:** Jessica Bon, Rehan Kahloon, Yingze Zhang, Jianmin Xue, Carl R. Fuhrman, Jiangning Tan, Mathew Burger, Daniel J. Kass, Eva Csizmadia, Leo Otterbein, Divay Chandra, Arpit Bhargava, Joseph M. Pilewski, G. David Roodman, Frank C. Sciurba, Steven R. Duncan

**Affiliations:** 1 Department of Medicine, University of Pittsburgh, Pittsburgh, Pennsylvania, United States of America; 2 Department of Radiology, University of Pittsburgh, Pittsburgh, Pennsylvania, United States of America; 3 Department of Surgery, Beth Israel Deaconess Medical Center, Harvard Medical School, Boston, Massachusetts, United States of America; 4 Department of Medicine, Indiana School of Medicine, Indianapolis, Indiana, United States of America; University of Montana, United States of America

## Abstract

**Rationale:**

Emphysema and osteoporosis are epidemiologically associated diseases of cigarette smokers. The causal mechanism(s) linking these illnesses is unknown. We hypothesized autoimmune responses may be involved in both disorders.

**Objectives:**

To discover an antigen-specific autoimmune response associated with both emphysema and osteoporosis among smokers.

**Methods:**

Replicate nonbiased discovery assays indicated that autoimmunity to glucose regulated protein 78 (GRP78), an endoplasmic reticulum chaperone and cell surface signaling receptor, is present in many smokers. Subject assessments included spirometry, chest CT scans, dual x-ray absorptiometry, and immunoblots for anti-GRP78 IgG. Anti-GRP78 autoantibodies were isolated from patient plasma by affinity chromatography, leukocyte functions assessed by flow cytometry, and soluble metabolites and mediators measured by immunoassays.

**Measurements and Main Results:**

Circulating anti-GRP78 IgG autoantibodies were detected in plasma specimens from 86 (32%) of the 265 smoking subjects. Anti-GRP78 autoantibodies were singularly prevalent among subjects with radiographic emphysema (OR 3.1, 95%CI 1.7–5.7, p = 0.003). Anti-GRP78 autoantibodies were also associated with osteoporosis (OR 4.7, 95%CI 1.7–13.3, p = 0.002), and increased circulating bone metabolites (p = 0.006). Among emphysematous subjects, GRP78 protein was an autoantigen of CD4 T-cells, stimulating lymphocyte proliferation (p = 0.0002) and IFN-gamma production (p = 0.03). Patient-derived anti-GRP78 autoantibodies had avidities for osteoclasts and macrophages, and increased macrophage NFkB phosphorylation (p = 0.005) and productions of IL-8, CCL-2, and MMP9 (p = 0.005, 0.007, 0.03, respectively).

**Conclusions:**

Humoral and cellular GRP78 autoimmune responses in smokers have numerous biologically-relevant pro-inflammatory and other deleterious actions, and are associated with emphysema and osteoporosis. These findings may have relevance for the pathogenesis of smoking-associated diseases, and development of biomarker immunoassays and/or novel treatments for these disorders.

## Introduction

Emphysema, defined as radiologic and/or histological evidence of lung parenchymal destruction, accounts for enormous world-wide morbidity and mortality [1]. Emphysema among tobacco smokers often occurs in association with chronic obstructive pulmonary disease (COPD), a complex syndrome typified by airway narrowing and inflammation, and diagnosed by the presence of expiratory airflow obstruction on spirometric testing [1]–[4]. Nonetheless, emphysema and COPD are by no means invariably concordant, and many patients severely afflicted by one of these lung abnormalities may have little or no evidence of the other [2]–[4]. In addition to directly attributable disability and premature deaths, smoking-associated lung diseases are also linked to many systemic abnormalities, including vasculopathies [5], lung cancer [6], renal dysfunction [7], and osteoporosis [8]. The abnormal and often pathological bone demineralization that occurs in smokers is particularly notable for being highly related to the presence and severity of emphysema, and is independent of the co-existence or magnitude of COPD *per se* [8].

While tobacco smoking is the single greatest risk factor for the development of lung disease in industrialized societies, additional mechanisms are also important, since only a fraction of heavy smokers have severe clinical manifestations [1]. Moreover, symptomatic, pathologic, and radiographic features are highly variable among afflicted individuals, familial clustering of cases is evident, and the disorders often progress despite smoking cessation [1], [4], [9], [10]. The pathophysiological processes that cause or promote emphysema and its co-morbidities remain enigmatic, although systemic immunological responses, including the actions of activated monocyte-lineage phagocytes, have been implicated in these disorders [11], [12].

Adaptive immune responses against a variety of autoantigens appear to be common among patients with lung disease attributable to smoking [13]–[15]. Unspecified autoantibodies from smokers have been shown to induce pulmonary epithelial cell cytotoxicity *in vitro*. [13] Immune complex and complement depositions are important mediators of autoantibody-induced tissue injury, and these abnormalities are also prevalent in the diseased lungs of smokers [13], [14]. Nonetheless, most reports of autoimmunity in patients with smoking-related lung disease to date are correlative rather than mechanistic. To our knowledge, moreover, no antigen-specific immune response has yet been implicated in the systemic co-morbidities of these pulmonary disorders [5]–[8].

Given the systemic nature of many immunological processes [16]–[19], we hypothesized autoimmune responses that could contribute to lung parenchyma destruction may also be involved in pathogenesis of one or more extrapulmonary co-morbidities that are associated with the emphysema. Accordingly, we conducted investigations to identify antigen-specific autoimmune responses relevant to both emphysema and osteoporosis in smoke-exposed subjects.

## Methods

### Subjects

Clinical and immunological correlation studies were performed in consecutive subjects with ≥10 pack-years of cigarette smoking, ages 40–79 years old, who were recruited under auspices of the Specialized Center for Clinically Oriented Research (SCCOR) registry at the University of Pittsburgh Medical Center. Subjects with unstable cardiovascular disease, malignancies, chronic oral steroid use, or BMI >35 were excluded. Each subject completed demographic and medical history questionnaires, and chest CT scans [6]–[8], [13]. Spirometry, lung volumes, and diffusion capacity (DLCO) were measured using standardized methods and reference equations. Healthy, never-smoked control subjects were recruited by solicitation of hospital personnel.

### Ethics Statement

The protocol was approved by the University of Pittsburgh Investigational Review Board. Each subject provided written informed consent.

### Emphysema Assessments

Chest CT examinations were performed with a General Electric (GE) LightSpeed VCT (64-detector) scanner at a radiation exposure of 100 mAs [6], [8]. A single expert radiologist, blinded to subject identities and other characteristics, interpreted the CT images using a validated [6]. 6-point semi-quantitative visual scoring system to define emphysema severity (0 = none, 1 = trace/minimal, 2 = mild, 3 = moderate, 4 = severe, 5 = very severe), corresponding to 0%, <10%, 10–25%, 26–50%, 51–75%, and >75% visual emphysema [6]–[8]. Our group has previously shown these visual emphysema scores are associated with clinically important outcomes in smokers [6]–[8], and the validity and comparability of these assessments with other radiographic measures of emphysema has also been established [2], [3].

### Blood Specimens

Plasma was obtained by centrifugation of heparinized phlebotomy specimens. Peripheral blood mononuclear cells (PBMNC) were isolated by density-gradient centrifugation [20]. Serum was obtained as the supernatant of clotted (non-heparinized) blood.

### Dual X-Ray Absorptiometry (DXA)

DXA measurements of bone mineral density (BMD) at the hip and lumbar spine were added to subject assessments after the initiation of enrollments in the SCCOR, and were available in the last 200 smoking subjects who provided plasma for immunological studies. Bone mineral density (BMD) was measured at the hip and lumbar spine using a Hologic 4500A Discovery bone densitometer and previously detailed methods [8]. BMD is reported as a T score, the number of standard deviations from young, gender and ethnic-specific reference means. T scores ≤−2·5 defined osteoporosis [8].

### Macrophages and Osteoclasts

CD14^+^ cells were isolated from PBMNC of normal, nonsmoking humans using anti-CD14 immunomagnetic beads (Miltenyi Biotec, Auburn, CA), and differentiated in the presence of M-CSF. In brief, cells were cultured for 7–10 days in complete RPMI with 10% FCS that was also supplemented with 50 ng/ml M-CSF (R&D Systems, Minneapolis, MN), and changed every 2–3 days. Flow cytometry confirmed >98% of the cells harvested ≥day seven expressed intracellular CD68^+^ and were CD3^−^ and CD19^−^.

Osteoclasts were derived from two healthy bone marrow donor volunteers. Mononuclear cells within the bone marrow aspirates were isolated by density gradient centrifugation and incubated in α-MEM with 20% FCS overnight at 37°C. Non-adherent cells were collected and cultured for 28 days in the same media supplemented with 25 ng/ml M-CSF and 30 ng/ml RANKL (R&D Systems), with frequent replenishment.

### Autoantigen Discovery

The nonbiased discovery assays that resulted in identification of autoreactivity to glucose regulated protein 78 (GRP78) among smokers with lung disease parallel those reported previously [21].

In brief, we hypothesized *a priori* that autoantigens associated with smoking-related emphysema could be identified by using patient-derived IgG antibodies to immunoprecipitate these autoantigens from cell lysates. The biologic validity of the putative autoimmunity could then be substantiated by demonstrating correlations between the presence of humoral autoreactivity to these self-proteins and disease prevalences. Additional evidence could be provided by finding concurrent T-cell autoreactivity, HLA bias, and discovering disease-relevant functional effects of the autoantibody(ies) [15]–[21].

Pooled circulating IgG antibodies isolated from six emphysematous subjects, known to have autoantibodies on previous study [13], were used to immunoprecipitate autoantigens from cell lysates. IgG from these subjects and another preparation from six normal control specimens were isolated by protein G, and then adhered to and covalently cross-linked to protein A columns (HP SpinTrap, GE Healthcare, Piscataway, New Jersey), per the manufacturer's protocol.

The cell lysates were preadsorbed with the normal IgG-protein A columns, and then applied to the emphysema IgG-protein A columns. After extensive washing, the putative IgG-bound autoantigens were eluted by acidification, pH neutralized, concentrated by centrifugal size-filtration (Millipore, Bellerica, MA), and identified by two dimension 10.5% sodium dodecyl sulfate polyacrylamide gel electrophoresis (SDS-PAGE). Gels were imaged by Typhoon TRIO (GE Healthcare) and analyzed by Image QuantTL software (GE Healthcare). Individual proteins were harvested by spot picking (Ettan Spot Picker, GE Healthcare), trypsin digested, and sequenced by matrix-assisted laser adsorption/ionization tandem time of flight mass spectrometry (MALDI-TOF/TOF) (Applied Biosystems, Carlsbad, CA).

Unpublished findings of previous investigations [13] had indicated the presence of an autoantibody with specificity for a then cryptic ∼75 kDa cell antigen tended to be associated with disease manifestations among smokers, and hence discovery of potential autoantigens of this ∼size was a particular interest.

Glucose regulated protein 78 (GRP78), a member of the heat shock protein 70 family, was identified in two sequential discovery assays. In addition to having an appropriate size, GRP78 seemed worthy of focus for additional study as a potential autoantigen in smokers given its myriad cellular functions [22]–[24] and role as an known autoantigen in other immunologic disorders [25], [26].

### Circulating Anti-GRP78 IgG

Immunoblots are a highly specific (“Gold Standard”) method for detection of antibodies [21]. These assays were performed using modifications of previously described methods. [21]

In brief, recombinant GRP78 (rGRP78) was purchased from Prospec (Rehovot, Israel). rGRP78 was prepared as a bulk solution and aliquots were frozen at −80°C until use. Volumes corresponding to two hundred and fifty (250) ng rGRP78 were concurrently added to multiple lanes of running gels (NuPage 4–12% Bis–Tris, Invitrogen, Carlsbad, CA) and electrophoresed. The proteins were transferred to nitrocellulose membranes and blocked with 5% dry milk in TTBS (50 mM Tris HCl [pH 7.4], 150 mM NaCl, 0.1% Tween 20). Membrane strips were separated by sectioning and each of these was individually incubated overnight at 4^o^ with a particular subject plasma specimen (@ 1∶20 dilution). All of the laboratory investigators performing these assays (RAK, JX, AB) concurrently incubated multiple subject plasma specimens, each with one of the individual membrane strips available from gels (plus positive and negative controls), as well as molecular weight markers, and were completely oblivious to subject identities or disease manifestations. Pilot study had shown that 1∶20 dilutions optimally distinguished emphysematous from normal populations, whereas more dilute specimens were too seldom positive in the disease subject specimens (and never positive among normal specimens). The strips were washed in TTBS, and then incubated for one hour with 1∶8000 dilutions of chicken anti-human IgG conjugated to horseradish peroxidase (HRP) (Thermo Scientific, Rockfort, IL). After another washing, HRP was detected by addition of Super Signal West Pico Chemiluminescent Substrate (Thermo Scientific), instantaneous exposure to radiographic film, and scored (positive or negative) by unanimous consensus of three investigators who were blinded to subject identities and clinical characteristics (Figure S1 in File S1). The few equivocal specimens (n<5) were repeated until all blinded judges were in agreement.

### Lung Specimens

Tissue (∼0.5–1 cm^3^) was dissected from emphysematous lungs explanted during therapeutic transplantations and cadaveric normal lungs that were not used as donor organs [20]. These specimens were fixed in neutral buffer Zn-formalin, paraffin embedded, and sectioned for immunohistochemistry (IHC) assays.

Bronchoalveolar lavage fluid (BALF) was obtained from lung explants by wedging sterile 5 mm plastic tubing in segmental bronchi, and successively infusing and aspirating 30 ml PBS aliquots using a syringe. BALF was centrifuged (400 *g*), and the supernatant was filtered (0.4 µm), concentrated using 3 kDa centrifugation-size filters (Millipore, Billerica, MA), and quantified by bicinchoninic acid (BCA) assay (Thermo Scientific). Alveolar macrophages within BALF were isolated by plastic adherence.

### Detection of GRP78 in Lung Specimens

Immunohistochemistry was used to assess *in situ* GRP78 expression in paraffin-embedded lung tissue sections [13], [21]. In brief, immunostaining was performed with a rabbit monoclonal antibody directed against Grp78 (Cell Signaling Technology, Danvers, MA) employing citrate antigen retrieval, as per the manufacturer's recommendation, biotinylated goat anti-rabbit IgG Jackson Immunoresearch West Grove, PA), and AB Complex HRP (Vector Laboratories, Burlingame, CA). Imaging methods have been previously detailed [13], [21].

GRP78 in BALF was detected by immunoblotting. Concentrated BALF (12 mcg protein) specimens were electrophoresed and processed as described above. Membranes were incubated with 1∶1000 dilutions of mouse anti-human GRP78 mAb (R&D Systems) at 4°C, followed by 1∶4000 dilutions of chicken anti-mouse IgG-HRP (Santa Cruz Biotechnology, Santa Cruz, CA). This immunoblotting method was validated using both commercial rGRP78 and lysates of normal human CD14^+^-derived macrophages (Figure S2 in File S1).

### Patient-Derived Anti-GRP78 Autoantibodies

IgG from pooled plasma specimens of six emphysema patients known to have anti-GRP78 autoantibodies (by prior immunoblot assays) was isolated by adherence to protein G. The IgG was applied to rGRP cross-linked to an AminoLink Plus Gel Spin column (Thermo Scientific), following manufacturer protocols. After extensive washing, anti-GRP78 was eluted by acidification, pH neutralized, concentrated using 5 kDa centrifugation filters and validated for IgG characteristics and specific avidity (Figure S3 in File S1). Eleven (11) ml plasma yielded 120 mcg of anti-GRP78 IgG. The endotoxin concentration within this autoantibody preparation was below the detection threshold of the limulus amebocyte lysate assay (LAL Chromogenic Endotoxin Quantitation Kit, Thermo Scientific), i.e., <0.1 EU/2 mcg of autoantibody.

### Indirect Immunofluorescence Assays (IFA)

Plate-bound normal alveolar macrophages from BALF (n = 3) and bone marrow osteoclasts (n = 2) were pre-incubated with 0.1 bovine serum albumin and azide-free Fc receptor blocker (Innovex Biosciences, Richmond, CA). After washing, the cells were incubated for 30 minutes with 0.5 mcg/ml of patient-derived anti-GRP78 IgG or the same concentration of pooled normal human IgG. After another washing, cells were incubated with FITC-conjugated anti-human IgG antibody (ImmunoConcepts, Sacramento, CA) and assessed by fluorescence microscopy, as detailed previously [13], [21].

### Macrophage Stimulation Assays

Media was removed from concurrent, autologous macrophage cultures, and the cells were incubated with 2 mcg/ml of either anti-GRP78 or normal human IgG for 30 minutes at 37°C. Media was then replaced, and both cells and supernatant were harvested after 18 hours.

Macrophages were suspended, fixed and permiabilized, and stained with anti-NFkB AlexFluor 647 (Cell Signaling Technologies, Danvers, MA), which has specificity for phosphorylated (Ser536) NFkB p65 subunits. Mean fluorescence intensities of the anti-GRP78-treated and normal IgG-treated macrophages were compared in ≥10,000 live cells, and analyzed using a BD FACSCalibur (BD Bioscience, San Jose, CA). Flow cytometry gates were established using control fluorochrome positive and negative macrophages (including isotype controls), as detailed elsewhere [20], [21].

Macrophage supernatants were analyzed for mediator productions using protein-suspended bead array platform multiplex kits (Bio-Rad, Hercules, CA), following the manufacturer's protocols [21].

### Bone Metabolites

Collagen type 1 C-telopeptide (CTX) was measured in sera by sandwich immunoassays using CTX reagents in an Elecsys 2010 (Roche, Indianapolis, IN), according to manufacturer protocols. Type 1 (N-terminal) Procollagen (P1NP) in plasma specimens was measured using a radioimmunoassay kit (Orion Diagnostica, Espoo, Finland).

### Human Leukocyte Antigen (HLA) Class II DRB1*15

Allele prevalence was determined by leukocyte DNA PCR-SSP [21]. Assessment of this particular allele was prompted by recent findings it is over-represented among idiopathic pulmonary fibrosis (IPF) patients with clinically-relevant autoimmunity [21].

### T-cell Functional Studies

PBMNC collected from consecutive SCCOR subjects were cultured for five days in complete media[13], [20], [21] in the presence of no added protein (baseline controls), or test antigens (i.e., rGRP78 or elastin split products [ESP]). ESP was purchased from Elastin Products Company (Owensville, MO). Both test antigens were boiled for 20 minutes and cooled on ice immediately prior to use, to obviate nonspecific mitogen effects, and added to cultures at final concentrations of 1 µg/ml. Pilot studies had shown there were no consistent relationships between test antigen concentrations ranging from 0.3 mcg-to-30 mcg/ml and assay results.

Proliferation was determined by incorporation of bromodeoxyuridine (BrdU), added two days prior to cell harvest, with measurements in gated CD4 T-cells established by flow cytometry, as detailed previously [20], [21]. Specific indices (SI) of proliferation were calculated as %CD4 T-cells that incorporated BrdU in cultures supplemented with the respective test antigen *minus* incorporation in concurrent unstimulated (control) cultures [20].

Intracellular IFN-gamma production in the last cohort of these culture specimens were also assessed by flow cytometry, using methods fully detailed previously [20], [21]. Specific indices (SI) of IFN-gamma production were calculated as %CD4 T-cells that produced this cytokine in cultures with added test antigen(s) *minus* cytokine production in concurrent unstimulated (control) cultures.

### Statistical Analysis

Ordered and continuous data were compared by Mann-Whitney tests. Wilcoxon signed rank tests compared results of two or more assays in the same specimen, with Bonferroni corrections for multiple comparisons. Dichotomous outcomes were analyzed by chi-square. Odds ratios (OR) and confidence intervals (CI) were calculated with univariate logistic regression. Multivariate logistic regression analysis was used to adjust for confounding factors. Alpha levels <0.05 were considered significant. All data was analyzed using SAS 9.2.

## Results

### Subjects

Characteristics of the smoke-exposure cohort are detailed in Table 1. The majority (79%, n = 209) of the 265 smoking subjects had one or more lung abnormalities (i.e., COPD and/or emphysema). One-hundred thirty-three (133), or 80%, of the 167 subjects who had expiratory airflow obstruction on spirometry (i.e., those who had COPD) also had emphysema. Conversely, forty-two (42), or 24%, of the 175 emphysematous subjects had normal spirometry. Another distinct cohort of healthy controls who had never smoked (n = 27) were 59±5 years old and 63% were male.

**Table 1:** Demographic and Clinical Characteristics of the Study Cohort.

	Aggregate	GRP78 Ab^−^	GRP78 Ab^+^	p value
Number	265	179	86	
Age (years old)*	67±0.4	67±0.5	67±0.7	NS
% male	58	63	47	0.01
Still smoking (%)	42	41	43	NS
Pack-years*	63±2	65±2	59±3	NS
ICS (%)	22	20	28	NS
Past oral steroids (%)	7	7	7	NS
FEV_1_% predicted*	77±1	78±2	74±3	NS
FEV_1_/FVC*	0.63±0.01	0.64±0.1	0.60±0.2	NS
DLCO% predicted*	68±2	71±2	64±2	0.02
Obstructed (%)	63	60	70	NS
Emphysema (%)	66	59	81	0.0003
Low Bone Density (%)	51	46	62	0.03
Osteoporosis (%)	9	4	18	0.002

All these subjects had ≥10 pack yr smoking histories. ICS  =  inhaled corticosteroid use; past oral steroids  =  oral steroid use within the preceding six months (none were currently taking oral steroids); FEV_1_% predicted  =  forced expiratory volume in the first second of expiration as percentages of predicted values; FEV_1_/FVC  =  ratio of FEV_1_ to forced vital capacity; DLCO% predicted  =  diffusing capacity for carbon monoxide as percentages of predicted values; Obstructed  =  subjects with spirometric findings of expiratory airflow obstruction (i.e., COPD); Emphysema  =  subjects with emphysema on CT scan; p values are for nonparametric comparisons of smokers who do not have anti-GRP78 IgG autoantibodies (GRP78^−^) vs. subjects who have these autoantibodies (GRP78^+^); NS  =  not significant. * denotes data are depicted as mean ± standard errors.

### Circulating Anti-GRP78 Antibodies

The prevalence of autoantibodies to GRP78 was greater in smokers (32.2%) than in the healthy, never-smoked controls (7.4%) (p = 0.007). The latter prevalence is similar to “false positive” rates of other disease-associated autoantibodies in healthy populations [13], [16], [20], [21].

Females were over-represented among the smokers who had anti-GRP78 autoantibodies (Table 1). Those who had anti-GRP78 autoantibodies also had decreased diffusion capacities, although their other pulmonary function tests, as well as medication use, were comparable to the autoantibody negative subjects.

HLA Class II DRB1*15 was under-represented among smokers with anti-GRP78 autoantibodies (22.4%) compared to the subjects who were autoantibody negative (34.7%) (OR 0.54, 95%CI = 0.3–0.99, p = 0.04). DRB1*15 prevalence in a local normal population was previously found to be 23% [21].

### Clinical Associations of Anti-GRP78 Antibodies

**Figure 1:**
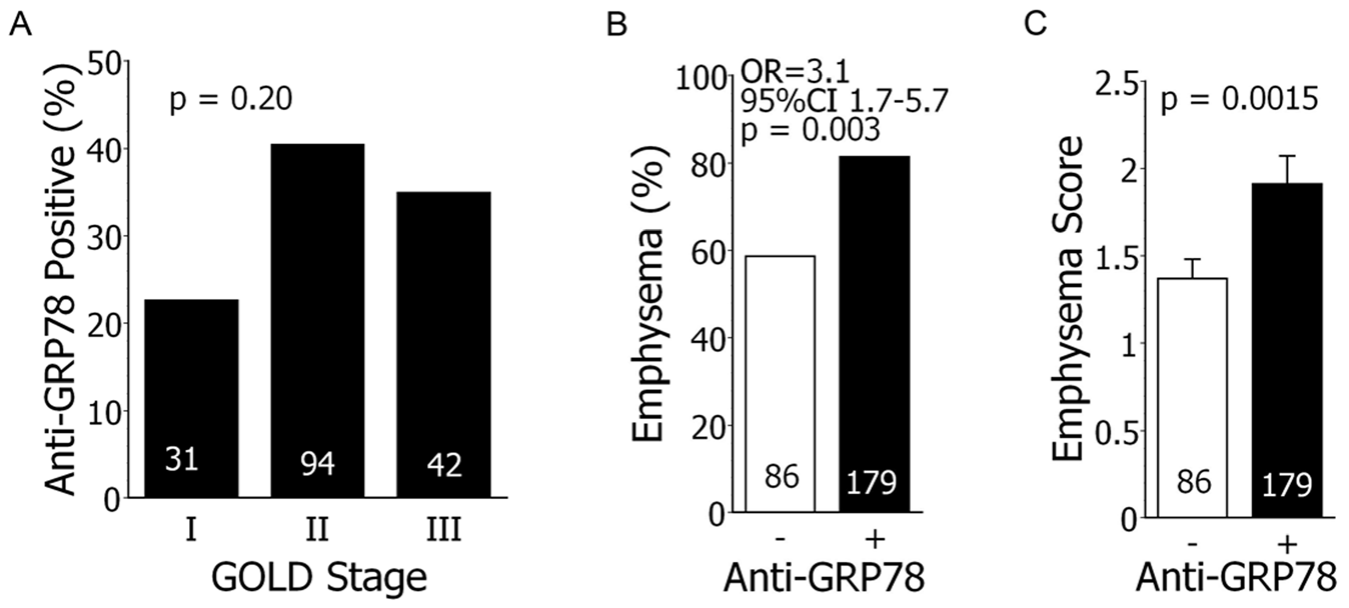
Anti-GRP78 autoantibodies, airflow obstruction, and emphysema. *A*.) Anti-GRP78 autoantibody prevalence was not significantly associated with COPD severity (GOLD stages) [40]. Numbers within columns denote subject n. *B*.) Anti-GRP78 autoantibodies are significantly associated with emphysema prevalence (%) in the smoking subjects. OR  =  odds ratio; CI  =  confidence interval. *C*.) Emphysema scores per CT were also significantly greater among those subjects with anti-GRP78 autoantibodies.

There were no significant differences of anti-GRP78 autoantibody prevalences in the smokers with COPD (35.9%) compared to those with normal spirometry (26.5%, p = 0.11), nor a significant correlation of this autoreactivity with the severity of airflow obstruction (Figure 1A).

However, the presence of anti-GRP78 autoantibodies was significantly associated with the prevalence (Figure 1B) and severity of emphysema (Figure 1C). Multivariate analyses showed that adjustment for gender and severity of expiratory airflow obstruction did not alter the relationship between anti-GRP78 positivity and emphysema (OR 3.1, 95%CI = 1.6–6.1, p = 0.001).

**Figure 2:**
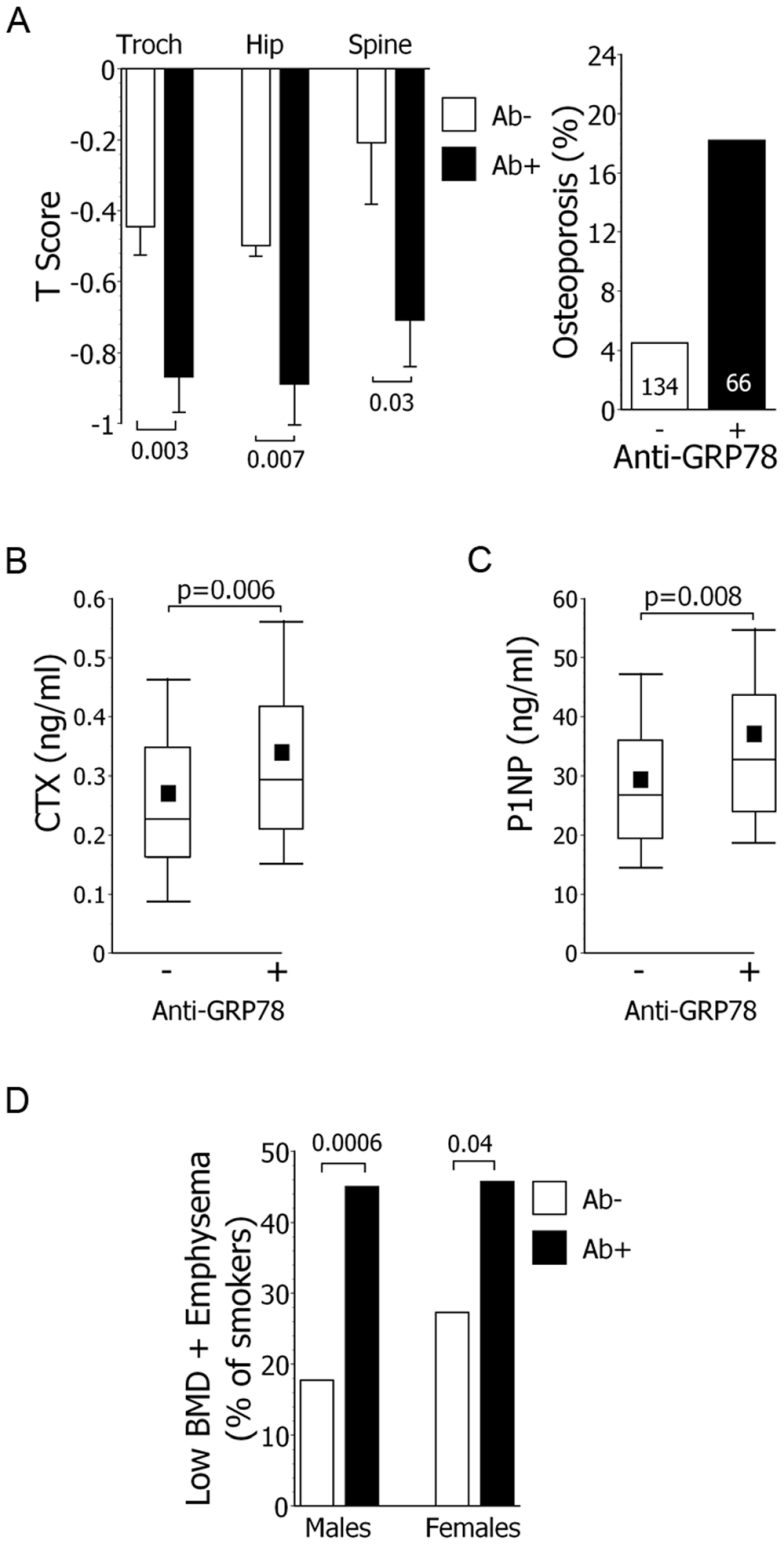
Anti-GRP78 autoantibody association with osteoporosis, bone metabolism markers, and concurrent low bone density and emphysema. *A*.) T scores (left panel) and osteoporosis prevalence (at either/both hip or spine) (right panel) among the smoking cohort. *B*.) Serum levels of bone turnover metabolite collagen type 1 cross-linked C-telopeptide (CTX) were greatest among smokers with anti-GRP78 autoantibodies. The lowest, second lowest, middle, second highest, and highest lines represent 10^th^, 25^th^, median, 75^th^, and 90^th^ percentiles, respectively. Means are denoted by solid squares. *C*.) Serum levels of bone turnover metabolite type 1 (N-terminal) procollagen (P1NP) were greatest among smokers with anti-GRP78 autoantibodies. *D*.) The relationship between GRP78 autoantibody positivity and the concurrent co-existences of low BMD and emphysema in smokers is significant in both genders, but greatest in males.

The presence of anti-GRP78 autoantibodies was also highly associated with decreased bone mineral density and osteoporosis (Figure 2A), and remained significant after multivariate adjustment for gender, airflow obstruction severity, tobacco burden, and steroid use (OR 4.2, 95%CI = 1.5–12.2, p = 0.008). Circulating metabolites of bone turnover were also greatest in the subjects with anti-GRP78 autoantibodies (Figures 2B,2C).

Given the associations of anti-GRP78 autoreactivity with emphysema and abnormalities of bone mineralization, a *post hoc* analysis was performed to compare autoantibody prevalence in those subjects who have **concurrent** emphysema and low BMD *vs*. the remaining smoking cohort. Anti-GRP78 autoantibody prevalence was significantly greater in the subjects who had **both** emphysema and osteopenia (50.7% *vs*. 25%, OR 3.1, 95%CI = 1.8–5.4, p<0.0001), and even more so among those with **both** emphysema and osteoporosis (80.0% *vs*. 29.6%, OR 9.5, 95%CI = 2.6–34.7, p<0.0001). A gender stratified analysis showed this relationship is strongest in males (Figure 2D).

### Intrapulmonary GRP78 Expression

**Figure 3:**
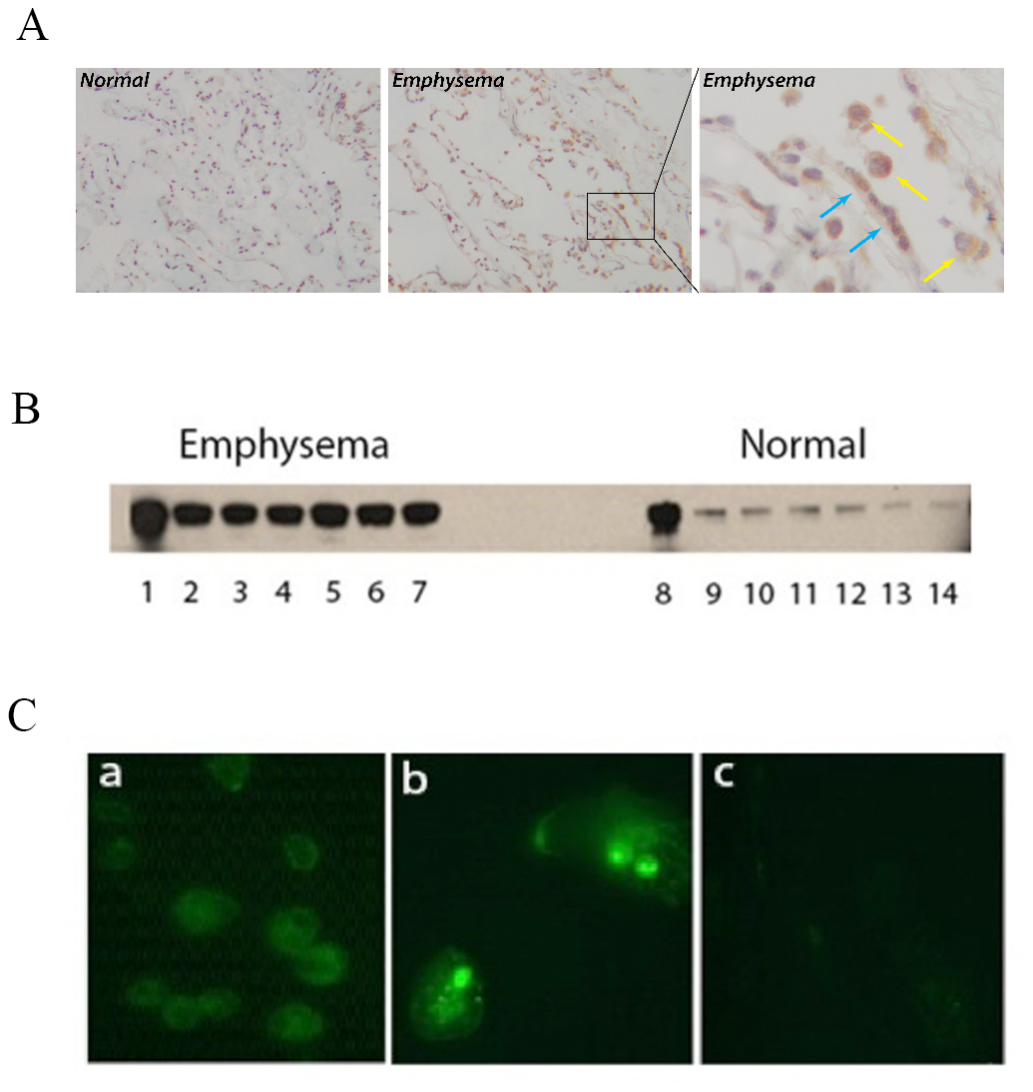
GRP78 expression in lung, bronchoalveolar lavage fluid, alveolar macrophages, and bone marrow derived osteoclasts. *A*.) Compared to normal lung (Left panel), emphysematous lungs (middle and right panels) demonstrated increased immunostaining in macrophages (yellow arrows) and alveolar epithelial cells (blue arrows). Magnification x100 left and middle panels, and magnification x400 in the right panel, n = 3. *B*.) GRP78 was also greater in bronchoalveolar lavage fluid (BALF) from emphysematous lungs compared to normal preparations. Lanes 1 and 8 are rGRP78 standards. Lanes 2–7 are BALF from individual emphysematous lung explants; lanes 9–14 are BALF from normal lung explants. All specimen lanes were loaded with equal amounts of BALF proteins. *C*.) Indirect immunofluorescent assays showed anti-GRP78 IgG isolated from patients bind to alveolar macrophages from normal lung explants (panel a), and osteoclasts derived from bone marrow (panel b). Normal human IgG control is illustrated in panel c.

GRP78 protein was and increased in emphysema specimens and predominantly localized in alveolar epithelial cells and macrophages (Figure 3A). GRP78 concentrations were also much greater in BALF from diseased lungs compared to the normal control preparations (Figure 3B).

### Anti-GRP78 Binding

In order to explore the possibility that anti-GRP78 autoantibodies have actions relevant to emphysema and osteoporosis, we first examined macrophage and osteoclast binding of anti-GRP78 IgG isolated from patient plasma. IFA showed the anti-GRP78 autoantibodies have avidity for these cells (Figure 3C).

### Cellular Effects of Anti-GRP78 Autoantibodies

**Figure 4:**
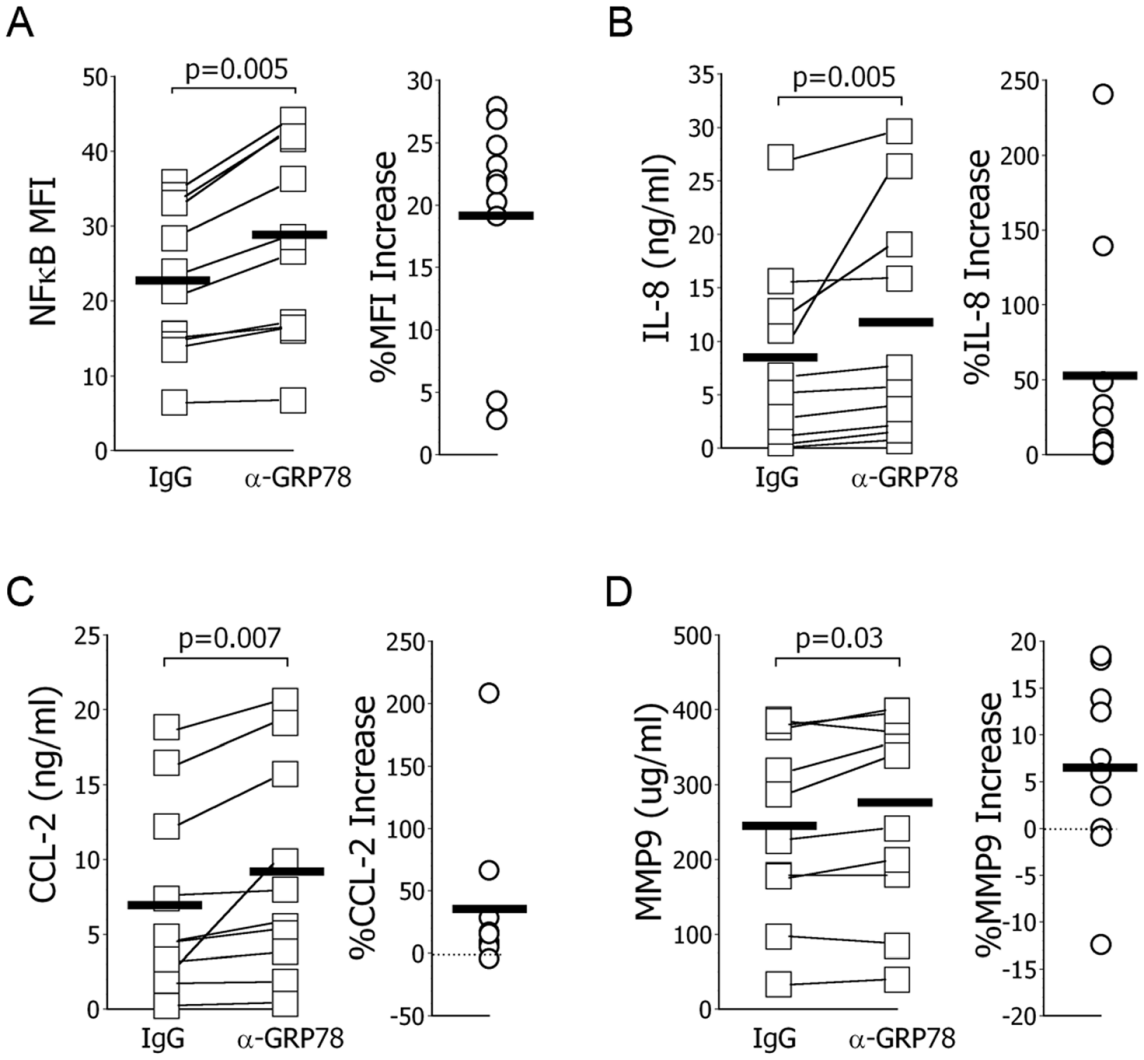
Cellular effects of autoantibodies to GRP78 on macrophages. *A*.) Mean fluorescence intensity (MFI) for phosphorylated NFkB among paired, concurrent autologous CD14^+^ derived macrophages was increased in all 10 normal specimens after incubation with patient-derived autoantibodies to GRP78 (α-GRP78), relative to control cells treated with normal human IgG. Patient derived anti-GRP78 autoantibodies also increased macrophage production of IL-8 (*B*.), CCL-2 (*C*.) and MMP9 (*C*.). Population means are denoted with a horizontal line.

To test for direct function-altering effects, patient-derived anti-GRP78 autoantibodies were incubated overnight with macrophages. These treatments resulted in increased macrophage NFkB activation (Figure 4A) and augmented productions of IL-8 (Figure 4B), CCL-2 (Figure 4C), and MMP9 (Figure 4D).

### CD4 T-cell Autoreactivity

**Figure 5:**
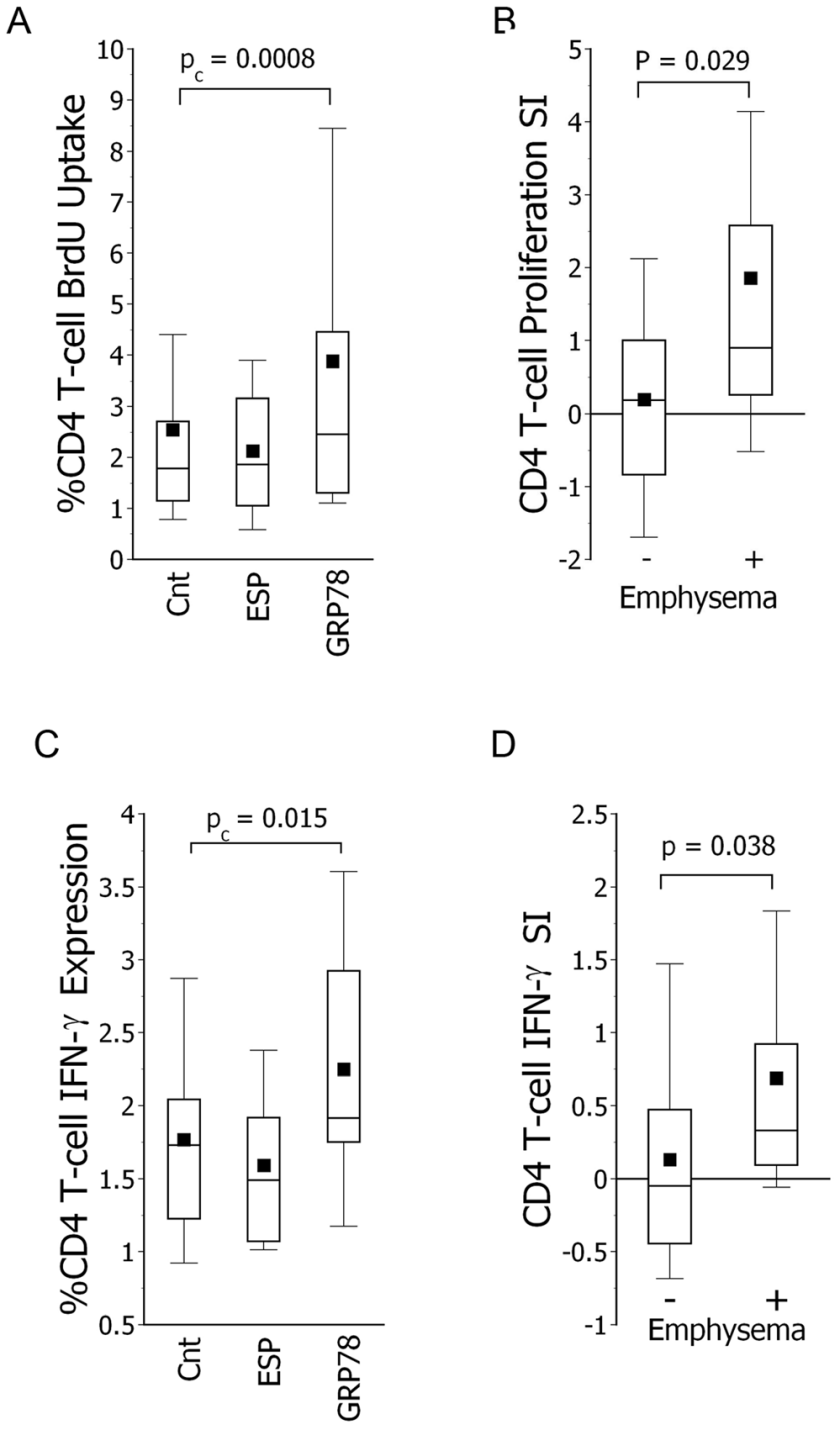
CD4 T-cell autoreactivity to GRP78. *A*.) Addition of GRP78 to PBMNC cultures increased CD4 T-cell proliferation (BrdU uptake) relative to media controls (no added protein) (n = 47), unlike addition of elastin split products (ESP). P_c_ denotes alpha level corrected for multiple comparisons. *B*.) GRP78-induced CD4 T-cell proliferation was greatest among cultures from smokers with emphysema (n = 34). Specific indices (SI) of proliferation were calculated as %CD4 T-cells incorporating BrdU in GRP78-supplemented cultures *minus* incorporation in concurrent media controls. *C*.) GRP78 also increased percentages of CD4 T-cells that produced IFN-gamma again, unlike effects of ESP. *D*.) IFN-gamma production was also greatest in the CD4 T-cells from the emphysematous smokers. Specific indices (SI) were calculated as %CD4 T-cells producing IFN-gamma production in GRP78-supplemented cultures *minus* that of concurrent controls.

Disease-associated autoantibody responses are also accompanied by concurrent T-cell responses to the autoantigen(s) [15], [16], [20], [21]. Addition of heat-denatured rGRP78 protein to PBMNC cultures from smokers induced proliferations of their CD4 T-cells, unlike effects of denatured ESP (Figure 5A). Moreover, the magnitude of the GRP78-triggered proliferative responses was greatest among the CD4 T-cells from emphysematous smokers (Figure 5B). CD4 T-cell IFN-gamma production was also uniquely increased in rGRP78-supplemented cultures (Figure 5C) and, again, especially so in the preparations from subjects with emphysema (Figure 5D). rGRP78 does not induce proliferation or IFN-gamma production in CD4 T-cells from normal nonsmoking subjects (data not shown) nor IPF patients [21].

## Discussion

Autoimmunity is a frequent complication of numerous, varied, primary injury responses [15]–[18]. The microbiome within smoke-damaged lungs is postulated to be highly immunogenic and, hence, a likely predisposition for the development of autoimmunity [13]–[15]. Neoantigens generated by reactive constituents within tobacco smoke can also provoke autoimmune responses [14]. Most “secondary” autoimmune responses are benign and clinically irrelevant. In some cases they are highly pathogenic and injurious, however, as exampled by carditis associated with microbial infections, neurological syndromes linked to malignancies, and myriad other tissue-specific autoimmune disorders [16]–[18], [26].

The adaptive immune responses to GRP78 in smokers have several features of classical autoimmunity [15]–[20]. The biological plausibility of a particular autoimmune response is conditional on the presence of the corresponding autoantigen in the target organ(s), and GRP78 is highly expressed in lungs, especially in the disease specimens (Figures 3A,3B). HLA haplotypes are major (and often the strongest) known genetic determinants of autoimmune susceptibilities. Finding distinct HLA alleles are over- or under-represented (conferring predilection or protection, respectively) within a defined disease population is a hallmark of autoimmune disorders [16], [21]. DRB1*15 appears to “protect” smokers against the production of disease-associated anti-GRP78 autoantibodies. This result is the obverse of analogous studies in IPF patients, in whom DRB1*15 is over-represented among those with autoantibodies against heat shock protein 70 (HSP70), a stress response protein with considerable sequence homology to GRP78 [21]. These disparate findings are thus indicative of biologically-distinct, antigen- and disease-specific autoimmune responses, rather than being a generalized epiphenomenon of “lung disease”.

Most compellingly, the increased prevalence of anti-GRP78 autoantibodies in emphysematous smokers is a defining criteria of “abnormal” autoreactivity [15]–[20]. The pathogenicity of anti-GRP78 IgG in smokers is at least implied by the stringent, independent, and overlapping associations of this specific autoantibody with concurrent emphysema, osteoporosis, and increased bone turnover. Furthermore, other findings here showing that patient-derived anti-GRP78 autoantibodies activate monocyte-lineage phagocytes, and enhance their productions of injurious mediators that are implicated in the genesis of emphysema and osteoporosis are direct evidences of deleterious autoantibody effects [11], [12], [27]–[29].

In particular, anti-GRP78 IgG treatments increased cellular elaborations of IL-8 and CCL2 (aka MCP-1), which are potent pro-inflammatory chemoattractants of neutrophils and monocytes/macrophages, respectively. Both of these mediators also stimulate osteoclastogenesis, and are increased among patients with osteoporosis and emphysema [11], [12]. Incubations with anti-GRP78 also increased macrophage production of MMP9, a Type IV collagenase produced by macrophages and osteoclasts that promotes enzymatic breakdown of extracellular matrix. MMP9 has an imputed causal or contributing role in lung parenchyma destruction, pulmonary metastases, and bone resorption [27]–[29].

Moreover, these varied effects occurred after only limited (18 hour) macrophage exposures to subphysiological concentrations (2 µg/ml) of the anti-GRP78 autoantibody. It seems possible that even greater effects might result from incubations with autoantibody concentrations found *in vivo* (∼11 µg/ml), and for longer periods. The cumulative effects of increased inflammation and protease activity in target tissues over many months-to-years may be especially deleterious, and emphysema and osteoporosis are typically most prominent among middle-aged or elderly smokers [1].

The *in situ* pathogenicity of GRP78 autoimmunity in smokers is also strongly supported by finding this stress response protein is an autoantigen of CD4 T-cells in these subjects, especially among those with emphysema. T-cells are inert to anatomically accessible self-constituents in healthy subjects, whereas overt reactivity of these lymphocytes to a protein that is abundant in diseased organs (Figures 3A,3B) is a “Gold Standard” of autoimmune disease [16], [19]. Antigen- (or autoantigen-) stimulated CD4 T-cells have protean and typically very injurious actions, including elaboration of numerous mediators that recruit and activate diverse leukocyte and somatic effector cells [21]. A finding of T-cell autoreactivity is very unlikely to be a benign epiphenomenon [16], [19]. Increased production of IFN-gamma in particular (Figures 5C,5D) is believed to be an important factor in the pathogenesis of smoking-associated lung disease [11]. Furthermore, the GRP78 reactivity of CD4 T-cells in emphysematous subjects cannot be simply attributable to global, nonspecific hyperactivity, since elastin split products (ESP) were inert (Figures 5A and 5C). Although ESP was previously reported to be an autoantigen of emphysema [30], we have been unable to replicate those findings, and similarly negative studies have been reported by other investigators [31]–[33].

GRP78 is also a frequent antigen of other autoimmune syndromes [25], [26]. GRP78 expression and extracellular export are up-regulated by varied stresses, including viral infections and smoke inhalation [22]–[24]. Ongoing immunologic responses that were initially directed at other antigenic peptides may generalize to co-associated chaperone proteins, such as GRP78, by a bystander mechanism and/or by epitope spread [25]. Subsequent injury (or infection) could cause further up-regulation of the newly antigenic GRP78, potentially creating a positive feedback loop that promotes immune responses and disease progression.

The specific mechanism(s) by which anti-GRP78 autoantibodies affect phagocytes is yet unknown, and is a focus of ongoing investigations in our laboratories. In addition to intracellular actions as an endoplasmic reticulum protein transporter and scavenger, and importance in the unfolded protein response, cell surface and extracellular GRP78 mediate anti-inflammatory and pro-resolutory effects [22]–[24]. Anti-GRP78 might counter these actions by decreasing the concentrations or bioavailability of immunomodulatory extracellular GRP78, and/or by activating signal transduction pathways after cross-linking cell surface GRP78. Anti-GRP78 autoreactivity is notably prevalent among subjects with rheumatoid arthritis, another smoking-associated disease, and the autoantibodies from these patients also enhance macrophage pro-inflammatory functions, again, by mechanisms still unknown [26].

The findings of GRP78 autoreactivity here lend support to an evolving paradigm of autoimmune pathogenesis in smoking-associated lung diseases [13]–[15]. The present studies show anti-GRP78 responses in smokers are manifest by activation of autoantigen-specific T-cells, a process that leads to numerous deleterious consequences [19], and anti-GRP78 IgG autoantibodies directly exert pro-inflammatory effects. Even if these pathogenic actions are completely discounted, the multiple strong clinical-immunological associations here indicate assays for specific autoimmune responses may be useful to identify smokers who are most at-risk for these interrelated disorders. Nonetheless, these findings need additional corroboration and refinement before the adoption of autoimmune assays into the clinical management of smoking patients.

In addition, many immune diseases, and antibody-mediated lung disorders in particular, are refractory to treatment with nonspecific immunosuppressive regimens (e.g., glucocorticoids). Conversely, however, specific therapies that directly target pathological autoimmune processes, including autoantibody removal or interruption of autoantibody production *per se*, more often have clinical efficacy [34]–[39]. The most important ultimate result of the present study and related reports may be to draw attention to the potential for novel treatments, mechanistically-focused at critical stages of autoimmune cascades, to prevent or slow progression of the morbid and refractory syndromes associated with smoking [15], [34]–[39].

## Supporting Information

File S1 Contains Figure S1, Immunoblot Detection of Anti-GRP78 Autoantibodies. Detection of circulating anti-GRP78 IgG. Immunoblots were used to detect circulating anti-GRP78 autoantibodies in plasma specimens See manuscript text for methodological details. *Left panel*: 75 kDa molecular weight marker (MW) and adjacent plasma specimen negative for anti-GRP78 (Lane A). *Right panel*: 75 kDa molecular weight marker (MW) and subject plasma specimen positive for anti-GRP78 IgG (Lane B). Note: GRP78 migrates on 12% Bis–Tris gels as though it were slightly smaller than its expected 78 kDa size. Figure S2, Detection of GRP78 Protein in Lung Specimens. Control immunoblots. *Lane A*.) rGRP detected with mouse anti-human GRP78 monoclonal antibody (R and D Systems) at 1∶500 dilution. The secondary antibody was chicken anti-mouse human IgG at 1∶4000 dilution. *Lane B*.) GRP78 in a human CD14^+^-derived macrophage lysate (29 µg total protein) was also detected using this anti-human GRP78 monoclonal antibody. MW.) 75 kDa molecular weight marker. Figure S3, Patient-Derived Anti-GRP78 Autoantibody Characterizations. Validations of patient-derived anti-GRP78 autoantibodies. *Left panel*: Evaluations of the patient-derived anti-GRP78 on SDS gels showed protein bands typical for IgG, i.e., 25 kDa light chains and 50 kDa heavy chains. *Right panel*: The avidity of the isolated, patient-derived autoantibody for GRP78 was confirmed by rGRP78 immunoblot. Lane A.) 75 kDa molecular weight marker; Lane B.) rGRP78. The patient-derived anti-GRP78 IgG was used here at a concentration of 1 µg/ml.
(DOCX)
