# Supplementary material for: Autoreactivity to Glucose Regulated Protein 78 Links Emphysema and Osteoporosis in Smokers
Source: PLoS One. 2014 Sep 12;9(9):e105066. doi: 10.1371/journal.pone.0105066 (PMC4162538; doi:10.1371/journal.pone.0105066)
Supplement: File S1 — Contains Figure S1, Immunoblot Detection of Anti-GRP78 Autoantibodies. Detection of circulating anti-GRP78 IgG. Immunoblots were used to detect circulating anti-GRP78 autoantibodies in plasma specimens See manuscript text for methodological details. Left panel: 75 kDa molecular weight marker (MW) and adjacent plasma specimen negative for anti-GRP78 (Lane A). Right panel: 75 kDa molecular weight marker (MW) and subject plasma specimen positive for anti-GRP78 IgG (Lane B). Note: GRP78 migrates on 12% Bis–Tris gels as though it were slightly smaller than its expected 78 kDa size. Figure S2, Detection of GRP78 Protein in Lung Specimens. Control immunoblots. Lane A.) rGRP detected with mouse anti-human GRP78 monoclonal antibody (R and D Systems) at 1∶500 dilution. The secondary antibody was chicken anti-mouse human IgG at 1∶4000 dilution. Lane B.) GRP78 in a human CD14+-derived macrophage lysate (29 µg total protein) was also detected using this anti-human GRP78 monoclonal antibody. MW.) 75 kDa molecular weight marker. Figure S3, Patient-Derived Anti-GRP78 Autoantibody Characterizations. Validations of patient-derived anti-GRP78 autoantibodies. Left panel: Evaluations of the patient-derived anti-GRP78 on SDS gels showed protein bands typical for IgG, i.e., 25 kDa light chains and 50 kDa heavy chains. Right panel: The avidity of the isolated, patient-derived autoantibody for GRP78 was confirmed by rGRP78 immunoblot. Lane A.) 75 kDa molecular weight marker; Lane B.) rGRP78. The patient-derived anti-GRP78 IgG was used here at a concentration of 1 µg/ml. (DOCX) [file pone.0105066.s001.docx]

**FILE S1**

**Immunoblot Detection of Anti-GRP78 Autoantibodies:** Immunoblots were used to detect circulating anti-GRP78 autoantibodies in plasma specimens (Figure S1).

**MW A MW B**


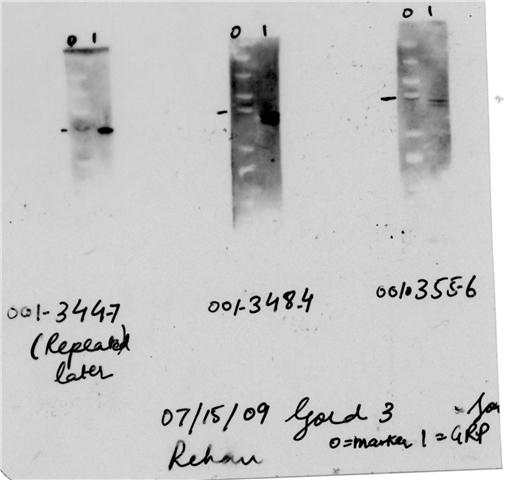

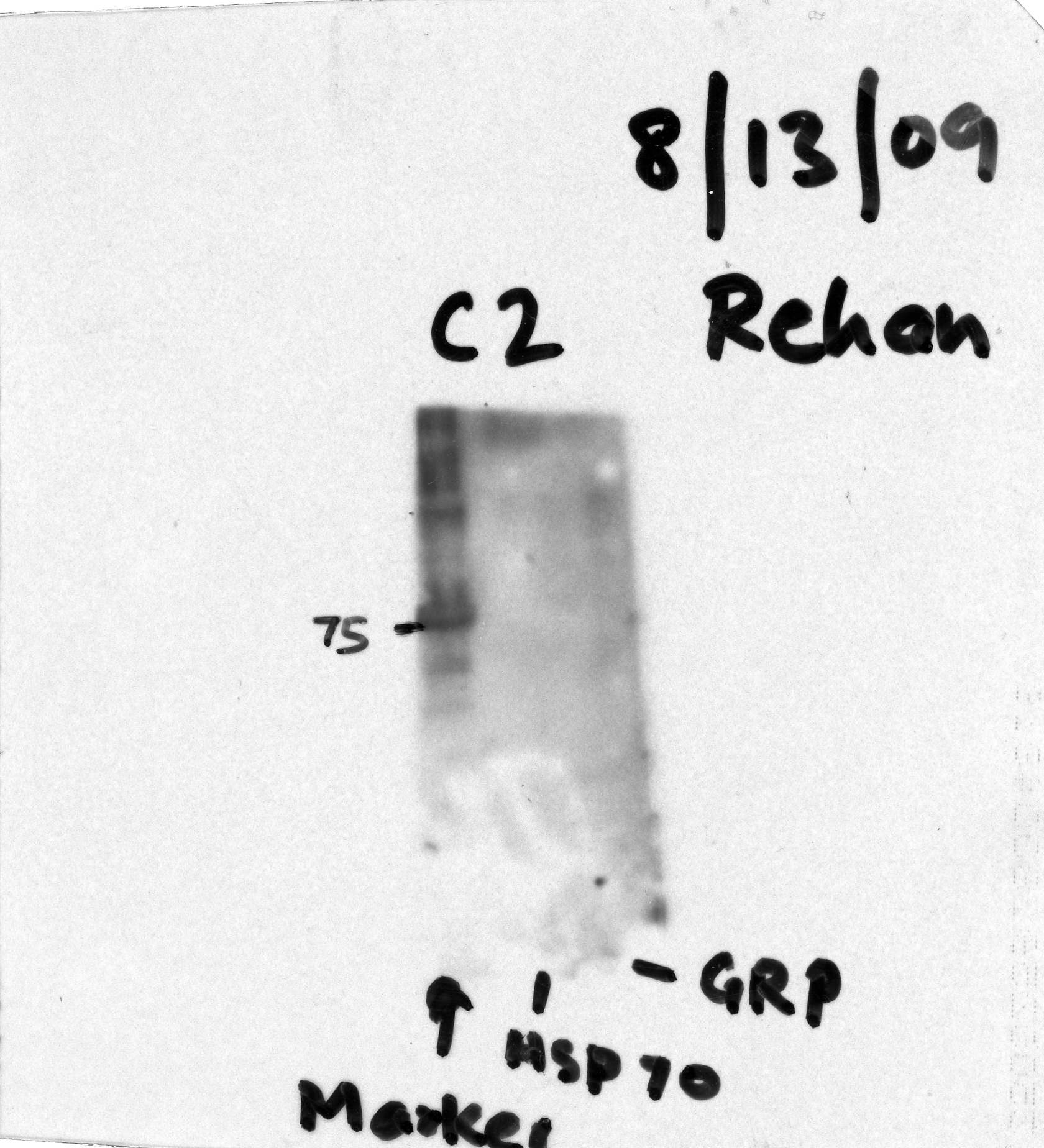


**Figure S1. Detection of circulating anti-GRP78 IgG**. See manuscript text for methodological details. *Left panel:* 75 kDa molecular weight marker (MW) and adjacent plasma specimen negative for anti-GRP78 (Lane A). *Right panel*: 75 kDa molecular weight marker (MW) and subject plasma specimen positive for anti-GRP78 IgG (Lane B). Note: GRP78 migrates on 12% Bis–Tris gels as though it were slightly smaller than its expected 78 kDa size.

**Detection of GRP78 Protein in Lung Specimens:**

A B MW


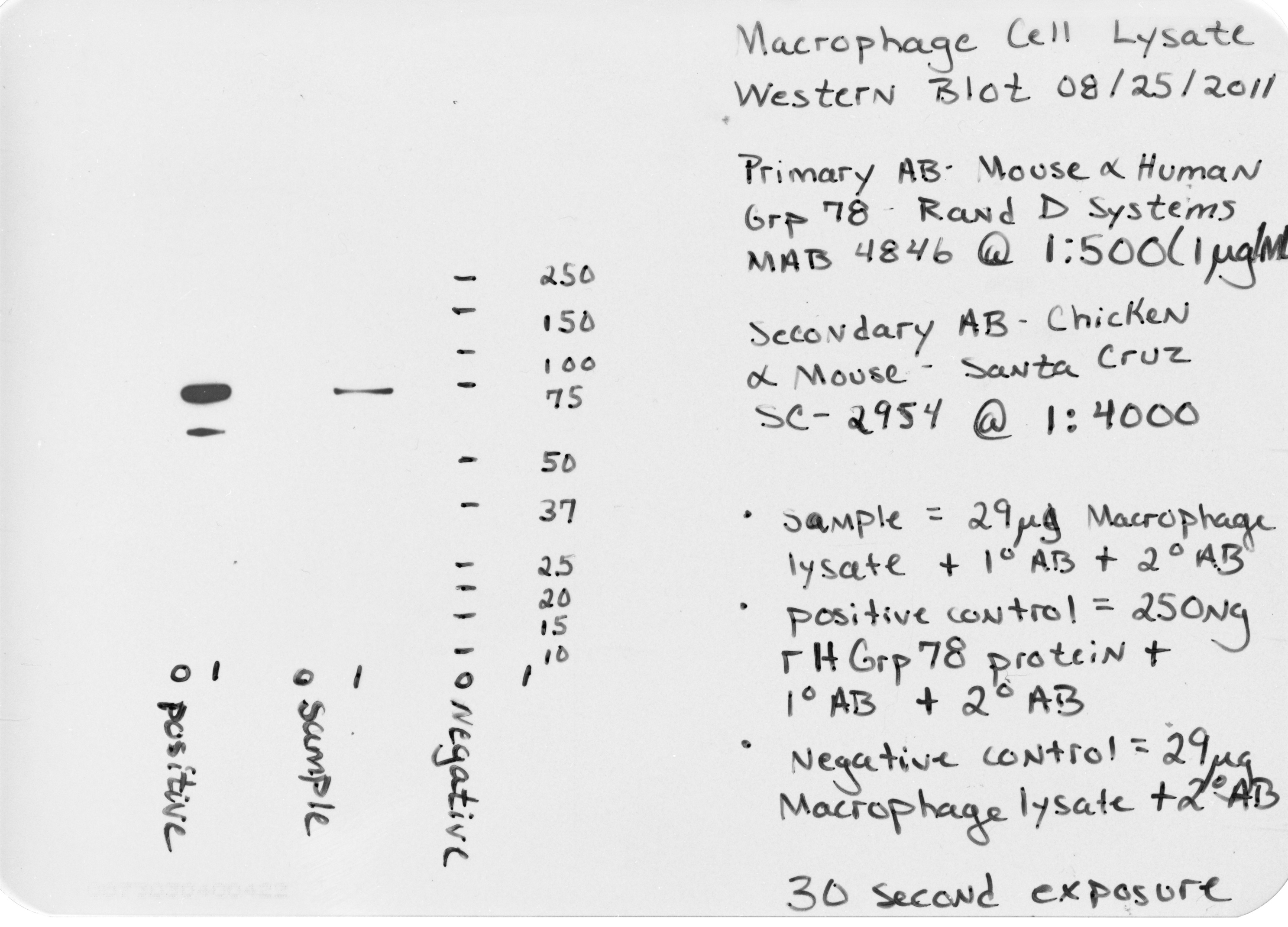


**Figure S2. Control immunoblots**. *Lane A.*) rGRP detected with mouse anti-human GRP78 monoclonal antibody (R and D Systems) at 1:500 dilution. The secondary antibody was chicken anti-mouse human IgG at 1:4000 dilution. *Lane B.*) GRP78 in a human CD14^+^-derived macrophage lysate (29 μg total protein) was also detected using this anti-human GRP78 monoclonal antibody. MW.) 75 kDa molecular weight marker.

**Patient-Derived Anti-GRP78 Autoantibody Characterizations:**


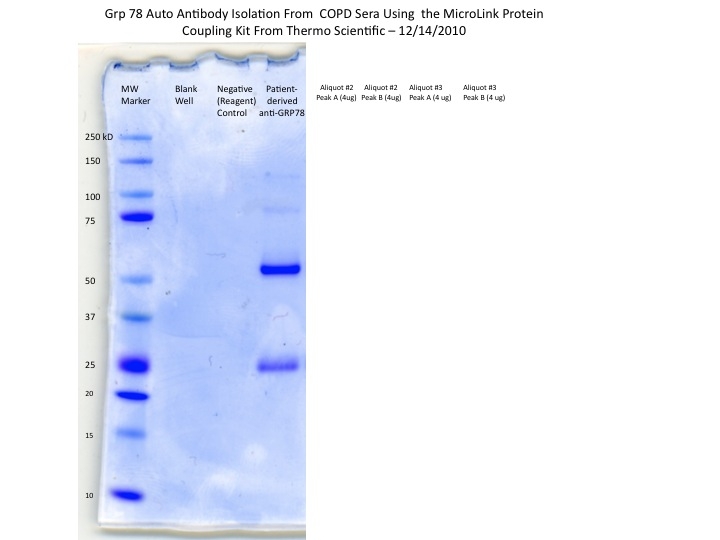


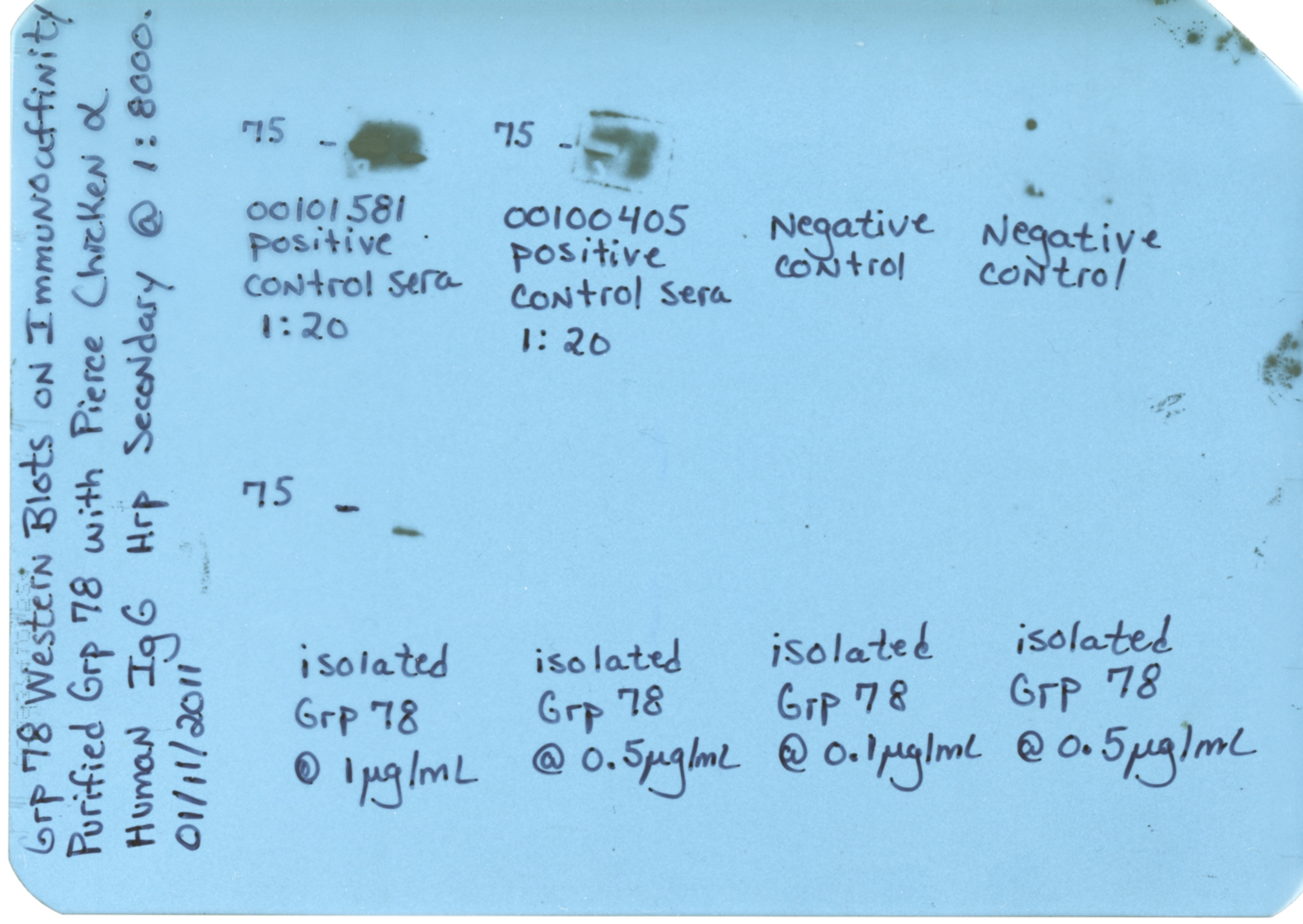
 **A B**

**Figure S3. Validations of patient-derived anti-GRP78 autoantibodies.** *Left panel:* Evaluations of the patient-derived anti-GRP78 on SDS gels showed protein bands typical for IgG, i.e., 25 kDa light chains and 50 kDa heavy chains. *Right panel:* The avidity of the isolated, patient-derived autoantibody for GRP78 was confirmed by rGRP78 immunoblot. Lane A.) 75 kDa molecular weight marker; Lane B.) rGRP78. The patient-derived anti-GRP78 IgG was used here at a concentration of 1 μg/ml.
